# The Relationship between Central Visual Field Damage and Motor Vehicle Collisions in Primary Open-Angle Glaucoma Patients

**DOI:** 10.1371/journal.pone.0115572

**Published:** 2014-12-29

**Authors:** Kenya Yuki, Ryo Asaoka, Kazuo Tsubota

**Affiliations:** 1 Department of Ophthalmology, Keio University School of Medicine, Tokyo, Japan; 2 Department of Ophthalmology, the University of Tokyo, Graduate School of Medicine, Tokyo, Japan; Harvard Medical School, United States of America

## Abstract

**Purpose:**

To investigate the relationship between visual field (VF) damage and history of motor vehicle collisions (MVCs) in subjects with primary open-angle glaucoma (POAG).

**Methods:**

MVC history and driving habits were recorded using patient questionnaires in 247 POAG patients. Patients' driving attitudes (carefulness) were estimated using Rasch analysis. The relationship between MVC outcomes and 52 total deviation (TD) values of integrated binocular VF (IVF), better and worse visual acuities (VAs), age and gender was analyzed using principal component analysis and logistic regression.

**Results:**

51 patients had the history of MVCs. Significant difference was observed between patients with and without history of MVCs only for: better VA, a single TD value in the superior-right VF, and the typical distance driven in a week (unpaired t-test, p = 0.002, 0.015 and 0.006, respectively). There was not a significant relationship between MVCs and mean deviation (MD) of IVF (p = 0.41, logistic regression). None of the principal components were significantly correlated with MVC outcome (p>0.05, polynomial logistic regression analysis). There was a significant relationship between IVF MD and Rasch derived Person parameter (R^2^ = 0.023, p = 0.0095). There was also a significant positive relationship between MVCs and the distance driven in a week (p = 0.005, logistic regression).

**Conclusions:**

In this study of POAG patients, MVCs were not related to central binocular VF damage. These results suggest the relationship between visual function and driving is not straightforward, and careful consideration should be given when predicting patients' driving ability using their VF.

## Introduction

Glaucoma is the second leading cause of blindness in the world, affecting approximately 60 million people [1]. It is a disease characterized by progressive retinal ganglion cell loss concomitant with peripheral and central visual field (VF) damage. Age is a significant risk factor for glaucoma [2]. The increase in the number of elderly people living in developed and developing countries combined with the fact that glaucoma is irreversible means there are an ever-growing number of drivers with glaucomatous VF defects.

Motor vehicle collisions (MVCs) are a serious public health concern. According to the United States Census Bureau, the number of police-reported traffic collisions exceeds 10,000,000 a year, and were the cause of 35,000 deaths in 2009 alone [3].

Many previous reports have investigated the relationship between visual function and MVCs [4]–[8]. Most of these studies analyzed the relationship between MVCs and summary measures, such as visual acuity (VA) and mean deviation (MD). However, it has been reported that specific VF regions are important for different tasks and affect hand-eye coordination [9], postural stability [10], risk of falling [4], and risk of fractures [11]. Indeed Murata et al. recently reported that interpreting point-wise VF sensitivity and VA together in the same statistical model resulted in a more accurate prediction of patients' disability in their daily lives [12]. Furthermore, Crabb et al. monitored patients' eye movements during driving simulations and reported that deterioration in the superior peripheral area of the binocular integrated visual field (IVF) could affect driving performance [13]. However, no study has investigated the relationship between point-wise VF sensitivities and history of MVCs in the real world. Thus, the aim of this study is to investigate IVF defects and their association with MVCs in subjects with primary open angle glaucoma (POAG).

## Methods

The study was approved by the Research Ethics Committee of the Keio University School of Medicine. Written informed consent was obtained from all subjects after explanation of the nature and possible consequences of the study. The study was performed according to the tenets of the Declaration of Helsinki.

### Study Design and Subject Enrollment

A total of 601 patients who visited Keio University Hospital (Tokyo, Japan), Iidabashi Eye Clinic (Tokyo, Japan) or Tanabe Eye Clinic (Yamanashi, Japan) between May 2011 and November 2011 were screened for eligibility by means of an ophthalmic examination that included slit-lamp biomicroscopy, funduscopy, gonioscopy, intraocular pressure measurements with Goldmann applanation tonometry, and VF examination with the Humphrey Field Analyzer (HFA, Carl Zeiss Meditec, Dublin, CA), using the 24–2 Swedish Interactive Threshold Algorithm Standard Strategy. Patients with ophthalmological diseases that could compromise VA or cause VF loss, such as cataract (except for insignificant senile change) were excluded. Patients with angle closure glaucoma, secondary glaucoma, age-related macular degeneration, diabetic retinopathy, and any fundus disease apart from POAG were also excluded. The eligible age was restricted to patients older than 40 years and less than 85 years. Of the 601 subjects screened, 230 were ineligible (S1 Table lists the reasons for exclusion). The purpose and methodology of the study were explained to every patient who met the inclusion criteria, and all patients agreed to participate. Answers to the driving questionnaire (details given below) were analyzed in a masked fashion, to avoid any observation bias.

### Diagnosis of POAG

POAG was diagnosed in 371 patients on the basis of the presence of the following three findings: (1) glaucomatous optic cupping represented by notch formation, generalized enlargement of cupping, senile sclerotic disc or myopic disc, or nerve fiber layer defects confirmed by glaucoma specialists (K.Y., and S.T.; see Acknowledgements) on fundus examination; (2) typical glaucomatous VF defects, such as Bjerrum scotoma, nasal step, or paracentral scotoma, compatible with optic disc appearance; and (3) an open, nonoccludable angle observed on gonioscopy.

### Questionnaire Regarding History of MVCs, Distance Driven in a Week and Attitudes towards Driving

All participants answered a questionnaire with the following questions (translated from Japanese):

(1) Do you have a driver's license? (Yes/No/Previously)

(2) How long have you driven/did you drive a car? (years)

(3) How far did you drive during the past one week? (km)

(4) Have you been involved in any traffic accidents, including single-car accidents or minor accidents, in the past five years? (Yes/No)

(5) Please circle any of the following driving situations that you avoid: at night (avoid night), in rain (avoid rain), in fog (avoid fog), on highways (avoid highways), high-speed driving (avoid high speed), lane changing (avoid lane change), driving close to the car in front (car distance). These questions were modified from the Driving Habits Questionnaire (DHQ) [14], [15]. Participants were also asked for their age and sex.

### Integrated Visual Field

A binocular IVF was calculated for each patient by merging a patient's monocular HFA VFs using the ‘best sensitivity’ method [16]–[19], where each TD value in the IVF is calculated using the maximum total deviation (TD) value (least negative) from each of the two overlapping points, as if the subject was viewing binocularly. IVF MD was calculated as the mean of all 52 TD values across the IVF.

### Statistical Analysis

Descriptive demographic statistics were calculated for the study patients. Age, better and worse VAs, and glaucoma severity defined by Mills glaucoma severity scale [20], and TD values of the 52 test points in the IVF were compared between patients with a history of MVCs and patients without a history of MVCs, using the non-paired t-test and chi square test.

The relationship between MVCs and IVF MD was investigated using Pearson's correlation. Then, in order to further analyze the relationship between MVCs and the VF in more detail, principal component analysis (PCA) was carried out to investigate the influence of all 52 IVF TD values, better eye and worse eye VAs, age and gender on MVCs. PCA developed by Karl Pearson in 1901 [21] is a statistical method to describe patterns of variation in multivariate data sets with correlated predictors. It has been reported that the sensitivities of VF test points in the central VF are strongly correlated with VA [22] and also that there is a close relationship between neighboring VF test points [23]–[25]. In PCA, observations from possibly correlated variables are analyzed as orthogonal ‘principal components’, which avoids the problem of multicollinearity [26]. In the current study, the number of meaningful principal components was decided based on the point at which the cumulative variance reached 90% [27]. Finally, the relationship between these principal components and the history of MVCs was investigated using a polynomial logistic regression model.

In addition, the attitudes of patients towards driving (the answers to question 5): “avoid night”, “avoid rain”, “avoid fog”, “avoid highway”, avoid high speed”, “avoid lane change”, “car distance” were analyzed using the Rasch model so that an index of what lengths a patient goes to in order to avoid traffic accidents could be derived (the ‘Person parameter’). Rasch Analysis is a special case of item response theory (IRT), whereby items and persons can be scaled according to a series of responses to different questions [28]. Rasch analysis places items and persons on a linear scale and provides the infit statistic to indicate how well different items describe the group of subjects and how well individual subjects fit the group [29], [30]. The infit statistic is calculated as mean square standardized residuals. An item infit less than 0.7 suggests redundancy, and values higher than 1.3 suggest unacceptable levels of noise in the responses and misfitting [31]; however, values between 0.5 and 1.5 may be considered productive for measurement [32]. Thus, the Rasch model provides a robust analysis of the validity of outcome measures [33], and indeed there are an increasing number of recent studies which have used Rasch analysis for testing instrument validity and applicability [34]–[41]. Unidimensionality was assessed using the 95% confidence interval (CI) of the residuals of PCA by carrying out bootstrapping (10,000 iterations). Unidimensionality indicates that a score produced by a measure represents a single concept [42] while multidimensionalilty indicates there is evidence of an additional component captured by the items [32], [43]. In PCA, an eigenvalue greater than 2.00 U is suggestive of a second construct being measured, indicating a multidimensional instrument [36].

Following construction of the Rasch model, the relationship between the Rasch derived-Person parameter and MVCs was investigated using logistic regression. Furthermore, the relationship between the Person parameter and a patient's IVF MD was analyzed using Pearson's correlation.

Finally, the relationship between the distance driven by a patient in a single week and history of MVCs was analyzed using logistic regression. The relationship between distance driven and the Rasch derived-Person parameter was also analyzed using Pearson's correlation.

All statistical analyses were carried out using the statistical programming language R (ver. 2.15.0, The R Foundation for Statistical Computing, Vienna, Austria) and Medcalc version 11.4.2.0; MedCalc statistical software, Mariakerke, Belgium). The R package ‘eRM’ was used to carry out analyses associated with the Rasch analysis, ‘stats’ was used to carry out PCA and the package ‘eigenprcomp’ was used to calculate the 95% CI of the PCA residuals. P values were adjusted for multiple comparisons using Benjamini's method [44].

## Results

Among 371 surveyed POAG patients, 73 patients did not have a driving license and 15 patients had given up driving (9 patients due to a fear of MVCs, 3 patients because of old age, 1 patient who had forgot to renew his/her driving license and a further 2 patients for unknown reasons). Of the remaining 283 driving patients, 247 supplied answers to the questions regarding MVC history and attitudes towards driving; the thirty-six subjects who did not supply answers were excluded because they did not actually drive. Subsequent analyses were therefore carried out using only data obtained from these 247 patients. The characteristics of these patients are summarized in Table 1. There were a large number of male subjects in our sample most likely reflecting the fact that more male Japanese drive.

**Table 1:** Subjects demographics.

247 POAG patients		
Gender (male∶female)	172∶75	
Age (years old) mean ±s.d. [range]	63.7±10.6	[40–84]
Better VA (LogMar) mean ±s.d. [range]	0.0034±0.017	[0.0–0.15]
Worse VA (LogMar) mean ±s.d. [range]	0.014±0.037	[0.0–0.15]
Better MD (dB) mean ±s.d. [range]	−2.6±3.9	[−20.1–2.2]
Worse MD (dB) mean ±s.d. [range]	−7.2±6.5	[−27.2–0.9]
IVF-MD (dB) mean ±s.d. [range]	−1.8±3.6	[−20.3–3.2]

Abbreviations.

POAG: primary open angle glaucoma, s.d.: standard deviation, VA: visual acuity, LogMar: the logarithm of the minimum angle of resolution, MD: mean deviation, dB: decibel, IVF: integrated visual field.

In the 247 studied patients, 51 patients experienced MVCs (‘MVC+’ group) and 196 patients had no history of MVCs (‘MVC-‘ group). The comparisons of age, better eye and worse eye VAs between these two groups are shown in Table 2. Comparisons of IVF TD values between the MVC+ and MVC- groups are shown in Table 3. Significant differences were observed only for better eye VA (unpaired t-test with Benjamini's correction for multiple comparisons, p = 0.032). There was not a significant relationship between MVCs and better eye VA, worse eye VA and IVF MD (logistic regression, p = 0.98, 0.24 and 0.41, respectively).

**Table 2:** Comparison of various visual function measures and person variables between the patients with and without a history of MVCs.

	MVC+ (mean ±s.d.)	MVC- (mean ±s.d.)	p value
Age	62.1±10.6	64.1±10.5	0.64
Better VA (LogMar)	0.000±0.000	0.004±0.020	0.036*
Worse VA (LogMar)	0.010±0.020	0.020±0.040	0.60
Gender (male∶female)	37∶14	135∶61	0.86
Distance driven per week (km)	148.2±204.6	73.2±121.2	0.14
IVF MD (dB)	−0.6±3.4	−0.8±3.7	0.67
Better MD (dB)	−3.0±3.8	−2.5±4.0	0.68
Worse MD (dB)	−8.1±7.3	−6.9±6.3	0.64
Better eye glaucoma severity# (0/1/2/3 or more)	6/37/5/3 (11.8%/72.5%/9.8%/5.9%)	40/129/18/9 (20.4%/65.8%/9.2%/4.6%)	0.86
Worse eye glaucoma severity# (0/1/2/3 or more)	1/24/12/14 (2.0%/47.1%/23.5%/27.4%)	5/107/48/36 (2.6%/54.6%/24.5%/18.3%)	0.86
Avoid night	12 (23.5)	64 (32.7)	0.64
Avoid rain	6 (11.8)	43 (21.9)	0.61
Avoid fog	7 (13.7)	32 (16.3)	0.86
Avoid highway	4 (7.8)	31 (15.8)	0.64
Avoid high speed	25 (49.0)	111 (56.6)	0.67
Avoid lane change	6 (11.8)	24 (12.2)	0.93
Car distance	25 (49.0)	90 (45.9)	0.86
Person parameter	−0.3±1.6	−0.6±1.5	0.61

# Glaucoma severity was categorized using Mills Glaucoma Staging system [20].

Chi square test was used for the variable of gender and unpaired t-test was used for other comparisons.

*represents p<0.05 with Benjamini's correction [44].

Abbreviations.

s.d.: standard deviation, VA: visual acuity, LogMar: the logarithm of the minimum angle of resolution, MD: mean deviation, IVF: integrated visual field, TD: total deviation, MVC: motor vehicle collision, Person parameter: Rasch analysis derived index of paying attention to avoid traffic accident.

**Table 3:** Exact IVF-TD values of MVC+ and MVC- groups.

		−0.5(2.4) −0.5(3.3) 0.98	−0.7(2.6) −0.6(3.7) 0.98	−0.7(2.7) −0.7(4.4) 0.98	−0.9(3.0) −1.5(5.8) 0.98		
	−0.8(3.4) −0.5(3.9) 0.98	−1.2(2.5) −0.8(3.9) 0.98	−9.9(12.7) −4.4(9.6) 0.32	−8.0(10.6) −6.8(11.2) 0.98	−2.2(4.6) −2.0(6.8) 0.98	−1.9(4.7) −1.7(6.3) 0.98	
−0.6(2.5) −0.4(3.4) 0.98	−0.8(3.0) −0.6(4.1) 0.98	−1.5(5.5) −0.8(4.4) 0.98	−0.8(3.6) −0.3(2.3) 0.98	−3.4(7.2) −2.4(6.5) 0.98	−2.6(6.7) −2.5(7.1) 0.98	−1.7(5.0) −2.3(7.1) 0.98	−1.8(4.3) −1.9(6.9) 0.98
−0.6(2.5) −0.5(3.6) 0.98	−1.6(3.4) −0.7(3.2) 0.98	−2.2(6.9) −1.2(4.9) 0.98	−0.2(2.3) −0.2(3.4) 0.98	−2.4(7.7) −1.8(6.3) 0.98	−4.1(8.9) −2.7(7.4) 0.98	−2.8(5.6) −2.8(7.4) 0.98	−1.6(4.9) −2.5(7.4) 0.98
−0.4(2.4) −0.3(3.6) 0.98	−1.4(3.5) −0.7(3.3) 0.98	−2.2(6.9) −1.0(4.3) 0.98	−0.3(1.8) 0.1(2.0) 0.98	−1.8(6.6) −1.3(5.4) 0.98	−4.4(9.2) −2.4(6.5) 0.98	−3.0(6.3) −2.6(6.9) 0.98	−2.3(5.5) −2.5(6.8) 0.98
−0.3(3.0) −0.1(3.7) 0.98	−0.6(2.5) −0.1(2.7) 0.98	−1.9(5.9) −0.7(4.0) 0.98	−0.8(2.1) −0.4(2.8) 0.98	−2.9(7.0) −2.1(6.5) 0.98	−3.5(8.3) −2.6(6.6) 0.98	−2.0(4.9) −2.2(6.0) 0.98	−1.6(4.5) −2.1(6.3) 0.98
	−0.3(2.3) −0.1(2.6) 0.98	−0.9(3.2) −0.4(2.8) 0.98	−8.0(12.2) −7.1(10.9) 0.98	−8.3(12.6) −7.9(11.3) 0.98	−2.3(5.2) −2.0(5.6) 0.98	−1.6(4.8) −1.5(5.2) 0.98	
		−0.5(2.4) −0.4(3.2) 0.98	−0.9(2.6) −0.6(3.5) 0.98	−1.8(5.5) −1.2(4.4) 0.98	−0.9(3.5) −1.4(5.0) 0.98		

Upper row in each grid represents mean (standard deviation) value in MVC+ group, middle row represents those in MVC- group and bottom row shows the p value obtained by comparing two groups using the unpaired t-test with Benjamini's correction [44].

Abbreviations.

IVF: integrated visual field, MVC: motor vehicle collisions.

IVF-TD, Integrated visual field-total deviation, MVCs, motor vehicle collisions.

In the PCA model, cumulative variance reached 90% with 17 principal components (see Table 4). None of these 17 principal components was significantly correlated with the MVC in the polynomial logistic regression analysis.

**Table 4:** Cumulative proportion of variance and the coefficients of variance of the principal components in the polynomial logistic analysis against a previous history of MVCs.

PCA Components	Cumulative proportion of variance	coefficients	p value
1	46.9%	0.01	0.82
2	58.9%	−0.06	0.30
3	64.5%	−0.11	0.33
4	68.9%	0.07	0.58
5	72.8%	−0.16	0.18
6	76.1%	0.04	0.76
7	78.3%	−0.10	0.53
8	80.2%	0.03	0.86
9	81.9%	0.31	0.09
10	83.5%	−0.25	0.15
11	84.8%	0.00	0.99
12	86.0%	0.15	0.47
13	87.2%	−0.01	0.98
14	88.2%	0.38	0.09
15	89.1%	−0.18	0.48
16	89.9%	−0.19	0.45
17	90.7%	0.28	0.29

Cumulative variance reached 90% with 17 principal components. None of these 17 principal components was significantly correlated with the MVC in the polynomial logistic regression analysis.

Abbreviations.

PCA: principal component analysis.

**Figure 1:**
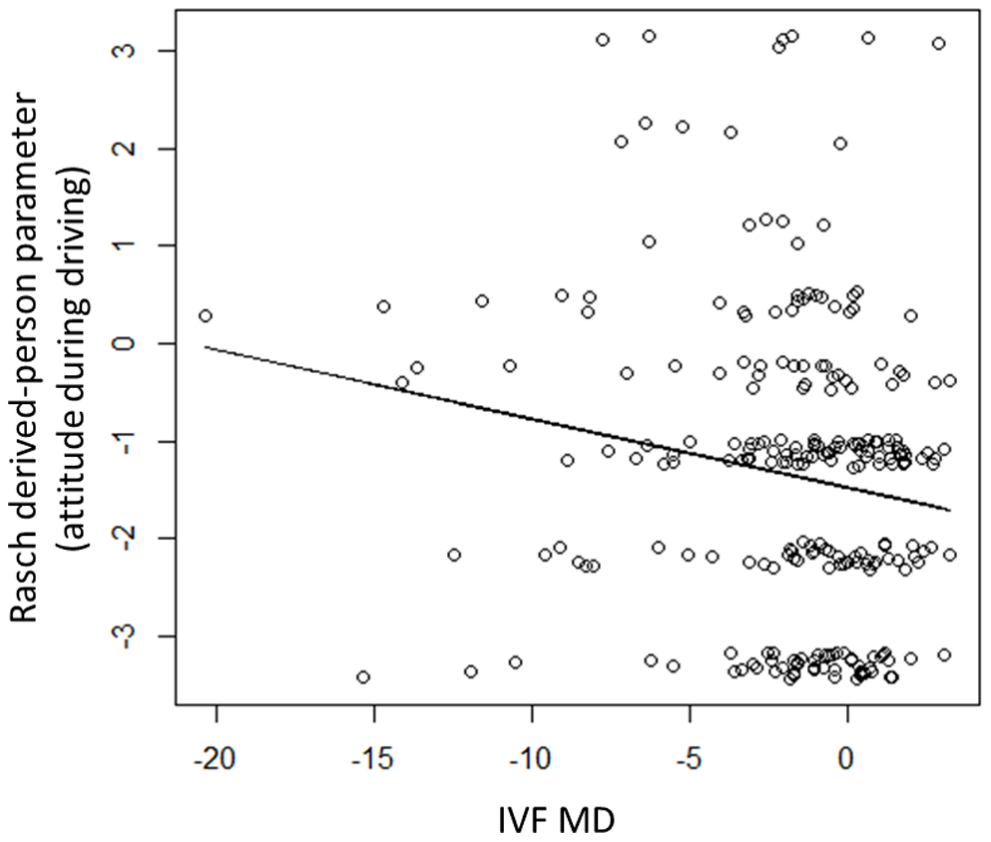
The relationship between the Rasch-derived person parameter (attitudes towards driving) and IVF MD. There was a significant relationship between the Rasch-derived person parameter (attitudes towards driving) and IVF MD (Y = −0.07*X −1.47, R^2^ = 0.023, p = 0.0095). A larger Rasch-derived person parameter value represents a more careful driving attitude. The ‘jitter’ function in the statistical programming language R (ver. 2.15.1, The R Foundation for Statistical Computing, Vienna, Austria) was used to aid interpretation of the scatter plot. Abbreviation. Rasch-derived person parameter: Rasch analysis derived driver's attitude based on the questionnaires of whether they avoid driving at night, in rain, in fog, on highways, high-speed driving, lane changing, driving close to the car in front. IVF MD: integrated visual field mean deviation.

All of the infit statistics associated with the Rasch analysis were constructive (values varied between 0.69 and 1.1). The eigenvalues of the PCA components obtained with the residuals of the Rasch analysis varied from 0.00002 to 2.1 and none of the lower CIs of these eigenvalues (0.02 to 1.7) exceeded the critical value of 2.0. There was not a significant relationship between MVC and the Rasch derived-Person parameters (logistic regression, p = 0.15). On the other hand, there was a significant relationship between the Rasch-derived person parameter (summarizing attitudes during driving) and IVF MD (Y = −0.07*X −1.47, R^2^ = 0.023, p = 0.0095), where a larger Rasch-derived person parameter value represents a more careful driving attitude; see Fig. 1.

There was not a significant relationship between the IVFMD and the distance driven by a patient in a week (Pearson's correlation, p = 0.87). MVC was significantly positively correlated with the distance driven by a patient in a week (logistic regression, p = 0.005). Conversely, there was not a significant relationship between the Rasch derived-Person parameter and the distance driven (Pearson's R^2^ = 0.009, p = 0.08).

## Discussion

In the current study, history of MVCs was investigated in 247 patients with POAG, according to driving attitudes and habits (including distance driven in a week) as well as IVF measurements. As a result, there was not a significant relationship between MVCs and IVF MD. In Japan, drivers drive on the left side of the road, however none of the TD values were significantly different between patients with MVCs and without MVCs. None of the principal components (derived from 52 IVF TD values, better and worse eye VAs, age and gender) were related to a history of MVCs. On the other hand, as we would expect, history of MVCs was significantly positively correlated with the distance a patient drives in a week. Patients' attitudes towards driving were analyzed using Rasch analysis. Interestingly, there was a significant relationship between the Rasch derived-Person parameter and IVF MD, suggesting that patients became more careful as their IVF progresses; however, there was not a significant relationship between the Rasch-derived Person parameter and the distance driven in a week.

Previous reports have investigated the relationship between MVCs and glaucoma patients' binocular VFs. Johnson and Keltner screened more than 10,000 subjects with automated perimetry, and reported that the accident rate in subjects with binocular VF damage was twice as high compared with age-sex matched controls (p<0.005), but was not significant higher in subjects with monocular VF damage (p>0.2) [45]. Also, Szylk et al. evaluated the association between driving performance using a driving simulator and peripheral VF damage, as measured with Goldmann kinetic perimetry. In their study, the horizontal extent of patients' binocular VFs had a significant correlation with simulator accidents (ρ = −0.47, p = 0.01); glaucoma subjects with at least 170 degrees of horizontal VF had no simulator accidents, while more than half of subjects with less than 170 degrees of horizontal VF experienced simulator accidents. No significant correlation was observed between accidents in the driving simulator and the horizontal extent of the VF in the better eye [46]. The fact that the findings in these previous studies are inconsistent with the results of our study could be attributed to the difference in the VF tests. Our study evaluated patients' central VFs using the 24–2 test pattern, on the contrary, Johnson et al. evaluated 100 degrees in the horizontal direction (40 degree on the nasal side and 60 degrees on the temporal side) while Szylk et al. used Goldmann perimetry. Thus, the current study suggests that a patient's binocular central IVF, obtained from 24–2 VFs is not related to MVC; however, in a different sample of patients with different VF defects, IVF damage may be associated with MVCs. Further studies should be carried out to investigate this possibility. Only static automated perimetry was measured in the current study as this is most frequently used in the current clinical settings, hence it was not possible to compare the current results with these previous studies. A future study in which Goldman perimetry and static automated perimetry measurements are included should be carried out so that the relevance of these measurements against MVC is compared.

In glaucoma, VF defects are usually primarily located in a superior or inferior hemifield, and hence there could be a “washout” effect due to the presence of significant VF defects that are in opposite hemifields between patients. Therefore, we divided the studied population into two groups: superior hemifield dominant and inferior hemifield dominant groups. Then, the relationship between MVC and 17 PCA components were analyzed using the generalized mixture logistic regression, instead of the polynomial logistic regression analysis, in which the effect of the different groups is considered. As a result, none of the PCA components were significantly related to MVC (p>0.05): results not shown. Similarly we divided the patients into four groups: superior-right, superior-left, inferior-right and inferior-left dominant groups, followed by the generalized mixture logistic regression for the four groups. Again none of the PCA components were significantly related to MVC (p<0.05): results not shown. Thus removing the ‘washout’ effect still did not reveal positive relationship between visual field data and MVC.

One explanation for finding no relationship between patients’ binocular central VFs and MVCs may be that patients can compensate for central VF damage during driving using eye movements. Crabb et al. investigated eye movements, using an eye tracker, in nine glaucoma patients (mean MD was −8.9 dB in the worse eye across all patients) and ten age-matched controls undergoing a driving hazard perception test (HPT) [13]. While undertaking the HPT, it was observed whether drivers slowed down or changed lanes when they realized a hazardous target, such as an oncoming cyclist or a pedestrian unexpectedly crossing the street. Crabb et al. found that the average duration of saccade eye movement and fixation was longer in glaucoma patients compared with the control subjects. They hypothesize that subjects with glaucoma use saccadic eye movement as a means of compensation for VF loss, as clearly demonstrated by an example case in the paper [13]. The study is supported by a different paper which also suggest eye movements compensate during driving [47]. Thus, it is possible that the glaucoma patients in our study use eye movements to compensate for VF damage, and as a result, do not experience a significantly larger number of MVCs.

Driving is clearly a complicated task, and is affected by many different things. Murata et al. evaluated the relationship between binocular VF test points and decreased vision related quality of life (QOL), and revealed that certain areas of the VF are more important for different tasks in daily life. For example, the peripheral superior and inferior areas of the left hemifield are more important for reading and writing, while the peripheral, mid-peripheral and para-central inferior regions tend to be more important for walking. Driving is obviously a very involved task – drivers frequently need to turn their heads, be aware of hazards as well as focus on their future direction [48]; age [49], physiological disabilities as well as mental status (such as drowsiness) will have an influence on driving performance. Moreover, drivers are aware that making a mistake during driving can cause an accident or even a fatality. Therefore, detecting hazards by saccadic eye- and head- movements [50] is much more important in driving than in other tasks such as reading and writing. Further studies should investigate the possibility that patients with glaucomatous VF damage attempt to avoid MVCs by driving more carefully than people without VF damage; such a study could be based on the experience of patients in a driving simulator.

The distance a patient drives per week was not associated with the Rasch derived-Person parameter. This is probably because there are many other confounding factors, such as subjects' occupations and access to public transportation. On the other hand, driving distance was significantly associated with the occurrence of MVCs. This would suggest that people do not decide the distance they drive based on their central binocular vision, and moreover that history of MVCs is simply a function of driving distance.

Patients' IVFs were constructed using the better sensitivity method [16]–[19]. In other words, a patient's worse VF sensitivity is ignored at all locations. Consequently the IVF may not be ideal for predicting MVCs in a given patient; some previous studies have suggested that there is a relationship between MVCs and the VF of a patient's worse eye, but not the VF of their better eye. McGwin et al. compared the prevalence of MVCs between 120 subjects with glaucomatous VF damage and 120 controls. In this study, they found that a severe VF defect (based on an AGIS score between 12–20) in the better eye did not significantly increase the risk of MVCs (odds ratio 3.2, 95% CI 0.9–10.4); however, in patients with even moderate VF defects (an AGIS score between 6–11), the worse eye significantly increased the risk of MVCs (OR 3.6, 95% CI 1.4–9.4 and OR 4.4, 95% CI 1.6–12.4, respectively) [8]. Furthermore, Tanabe et al. showed that in POAG subjects with severe VF defects in the worse eye (MD less than −10 dB), there was approximately a nine times increase in the likelihood of MVCs [51]. In addition, Haymes et al. evaluated driving performance in glaucoma subjects and controls based on a real-world driving exercise (under the supervision of a licensed driving instructor). In this study, worse eye MD was significantly associated with the instructor's overall rating of driving performance (r = 0.66, p = 0.002); however, better eye MD (r = 0.41, P = 0.08) and HFA binocular Estermann score (r = 0.30, p = 0.019) were not significantly associated with the overall rating [6]. Indeed, binocular visual summation is a result of binocular rivalry in which visual cortex perception is longer in the dominant eye [52], which is not considered in the simple ‘best sensitivity’ IVF model [16]. Nevertheless, the IVF is an established method for predicting binocular sensitivity [17] and has been used in many previous studies [53]–[58]; it may be appropriate to apply this model in ‘non-urgent’ tasks in daily life, such as reading and writing. However, the influence of the non-dominant eye requires further investigation; it may be associated with worse sensitivity in the binocular comparison, delaying visual perception in ‘dynamic’ tasks, such as driving. Nonetheless, there was not a significant difference in the distribution of glaucoma severity of better and worse eyes in the current study.

There was a significant difference in the better eye VA between the MVC+ and MVC- groups; however, logistic regression analysis did not suggest a significant relationship between better eye VA and MVCs. Thus, the risk of MVCs does not increase in proportion to the deterioration in better eye VA. Furthermore, none of the principal components from PCA (which included better eye VA) were significantly related to the MVC outcome. It should be noted, however, that most patients' VAs were preserved in the current study; hence further investigations should be carried out to explore the relationship between better eye VA and MVCs, such as seeking a cut-off value of better eye VA.

One of the clear limitations of our study was the reliance on self-reported patient interviews about their history of MVCs, especially because this may have resulted in recall bias [59]: glaucoma patients who have been followed for a long time by the same doctor may hesitate to provide a full history of MVCs. However, we would expect that this influence would be somewhat limited as Marottoli et al. have reported that self-reported MVCs provide sufficient information to assess car accidents, when compared to state-reported MVCs [60]. Another limitation of our study is that visual function data were obtained after MVCs had occurred; the longest possible interval between visual function data and MVCs is 5 years, which is sufficiently long that subjects' VF defects may have worsened during that period.

## Conclusions

In this study, MVCs were not related to POAG patients' central binocular visual fields. This result suggests that the relationship between visual function and driving is not straightforward and careful consideration should be given when predicting patients' driving ability from their IVF, especially when revoking driving licenses from occupational drivers and from patients who live in areas where there is no alternative transportation.

## Supporting Information

S1 Table **Reasons for excluding.** Abbreviation: POAG: primary open angle glaucoma.
(DOC)

S1 Data **Analyzed data (xlsx).**
(XLSX)
