# Supplementary material for: The Relationship between Central Visual Field Damage and Motor Vehicle Collisions in Primary Open-Angle Glaucoma Patients
Source: PLoS One. 2014 Dec 29;9(12):e115572. doi: 10.1371/journal.pone.0115572 (PMC4278721; doi:10.1371/journal.pone.0115572)
Supplement: S1 Table — Reasons for excluding. Abbreviation: POAG: primary open angle glaucoma. (DOC) [file pone.0115572.s001.doc]

Table 1. Reasons for excluding subjects from the study

| Reasons | POAG subjects (no.) |
| --- | --- |
| Younger than 40 | 28 |
| Older than 85 | 25 |
| Unable to walk unassisted | 0 |
| Refusal to participate | 10 |
| Dementia | 3 |
| Low visual acuity | 24 |
| Secondary glaucoma | 62 |
| Primary angle-closure glaucoma | 16 |
| Post retinal detachment | 21 |
| Diabetic retinopathy | 36 |
| Bullous keratoplasty | 2 |
| Age-related macular degeneration | 2 |
| Retinitis pigmentosa | 0 |
| Other ocular diseases | 1 |
| Total | 230 |
